# Rare uro-genital manifestations of von Recklinghausen disease: Scrotal, penile, and intrapelvic involvement with bladder and spermatic cord extension: A case report

**DOI:** 10.1016/j.radcr.2025.03.008

**Published:** 2025-04-05

**Authors:** Nadia El Mahi, Amal Mojahid, Hajar Siouri, Hamid Ziani, Siham Nasri, Imane Kamaoui, Imane Skiker

**Affiliations:** Department of Radiology, Mohammed VI University Hospital, Faculty of Medicine, University Mohammed First, Oujda, Morocco

**Keywords:** Child, Von Recklinghausen, Scrotum, Penis, Bladder, Spermatic cord, Ultrasound, MRI

## Abstract

Neurofibromatosis is a group of genetic disorders comprising 2 main types: type 1 neurofibromatosis (NF-1) and type 2 neurofibromatosis. The most common form is NF-1, also known as Von Recklinghausen disease. The clinical manifestations and presentation of this condition are variable. In this report, we present a unique case of a 10-year-old child with NF-1 who presented with scrotal swelling associated with progressive penile enlargement. After a thorough evaluation, the diagnosis of a plexiform neurofibroma was made. MRI evaluation revealed an intrapelvic extension of the tumor affecting the bladder and spermatic cords. This rare clinical presentation makes this case particularly interesting and highlights the diverse manifestations of Von Recklinghausen disease.

## Introduction

Type 1 neurofibromatosis (NF1) is a complex multisystem phacomatosis, inherited in an autosomal dominant manner, characterized by both cutaneous and noncutaneous symptoms [1], with a prevalence of approximately 1 in 3,000 and variable clinical expressivity. However, disease penetration is almost complete, reaching nearly 100% by the age of 5, meaning that most individuals carrying the genetic mutation will develop clinical manifestations in early childhood [2]. It is typically characterized by the presence of café-au-lait spots, Lisch nodules, and neurofibromas. Although these tumors can develop in any region of the body, primarily in the head and neck, pelvis, and limbs [3], they rarely localize to the urogenital area. However, urogenital involvement in Von Recklinghausen disease remains infrequent, particularly those simultaneously affecting the scrotum, penis, bladder, and spermatic cords, as discussed in this article, making this study particularly remarkable and exceptional.

## Case report

This is a 10-year-old boy presenting with scrotal swelling for 2 years, initially noticed by his parents. The swelling gradually increased over time. No similar swelling was noted elsewhere on his body. This swelling did not cause any urinary difficulty, and the child exhibited no other symptoms such as constipation, abdominal distension, palpable mass, urinary symptoms, bone or joint pain, seizures, anorexia, weight loss, visual disturbances, or hearing loss. Physical examination revealed an otherwise healthy boy.

Genitourinary examination showed an abnormally enlarged penis with slightly thickened skin from the base to the tip. Both corpora cavernosa, the glans, and the urethra were palpable and not fixed to the thickened skin of the penis, with the testes in their respective scrotal sacs. The scrotum was also thickened, and the proximal limit of this thickening could not be reached, suggesting that the thickening was not limited to the scrotum and could extend deeper.

Four hyperpigmented skin macules (café-au-lait spots) were also present on the body, the largest of which measured 4 cm on the back. Family history revealed similar cutaneous signs in the mother and younger sister, both of whom appeared healthy.

**Fig. 1:**
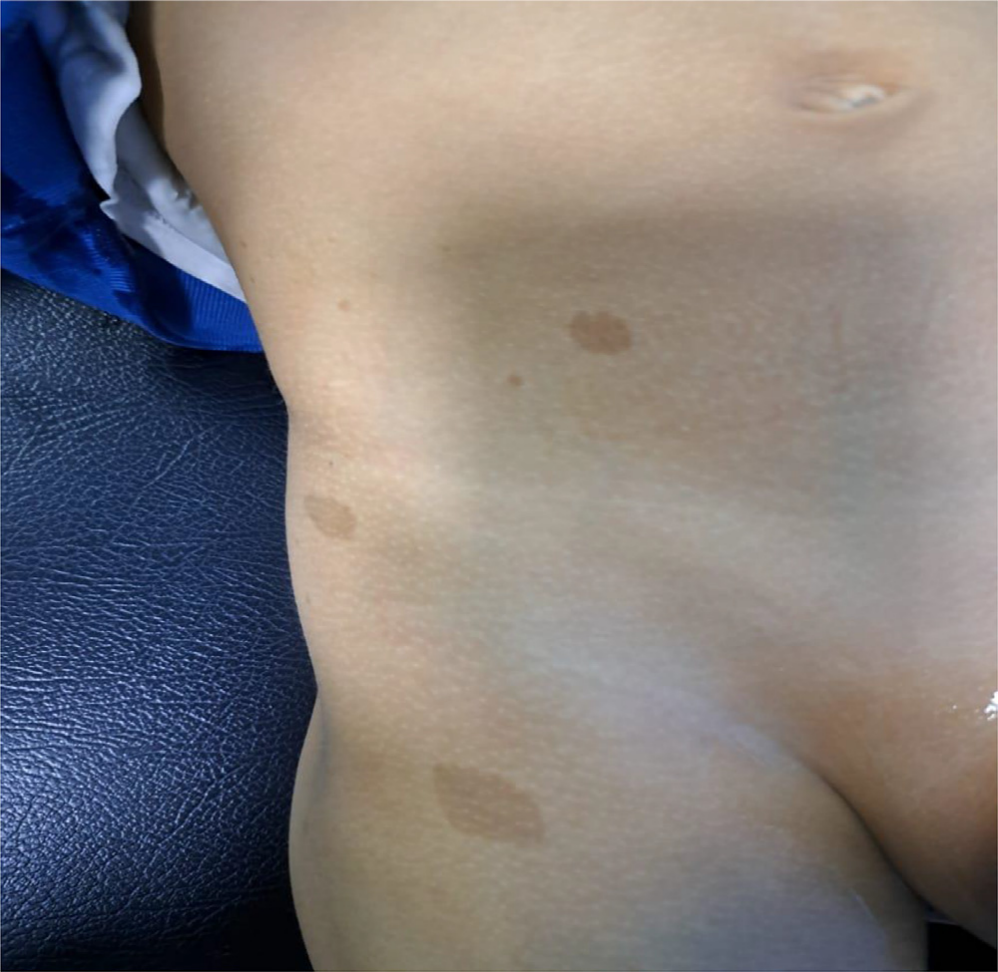
Hyperpigmented skin macules (café-au-lait spots).

Complete blood work and renal function tests were normal. Urinalysis and urine culture were negative. Visual field assessment was normal. Abdominal and testicular ultrasound (Fig. 1) revealed:

• At the scrotal level, bilaterally in the paramedian position, a well-defined, hypoechoic, heterogeneous oval formation with a microcystic appearance, crossed by horizontal trabeculae and vascular structures.
• Bilateral hypertrophy of the spermatic cords, with heterogeneous hypoechoic content containing nonvascularized cystic formations on color Doppler.
• The bladder showed regular and diffuse wall thickening, not visualized on color Doppler; the kidneys, ureters, and bilateral upper urinary tract were normal.

A pelvic MRI (Fig. 1) was indicated to assess the extent of this formation, showing diffuse intrapelvic infiltration with isosignal on T1, intermediate signal on T2, and a microcystic appearance, with heterogeneous enhancement following gadolinium injection, causing a mass effect on surrounding organs. This infiltration extended into the extratesticular scrotal envelopes, infiltrated the membranous and spongy urethra, and separated the corpora cavernosa, resulting in the thickened appearance of the penis. The spermatic cords were enlarged, with diffuse and homogeneous infiltration showing high T2 signal. Diffuse and homogeneous bladder wall thickening was also noted.

Given the café-au-lait spots and MRI findings, a diagnosis of urogenital NF1 was considered.

**Fig. 2:**
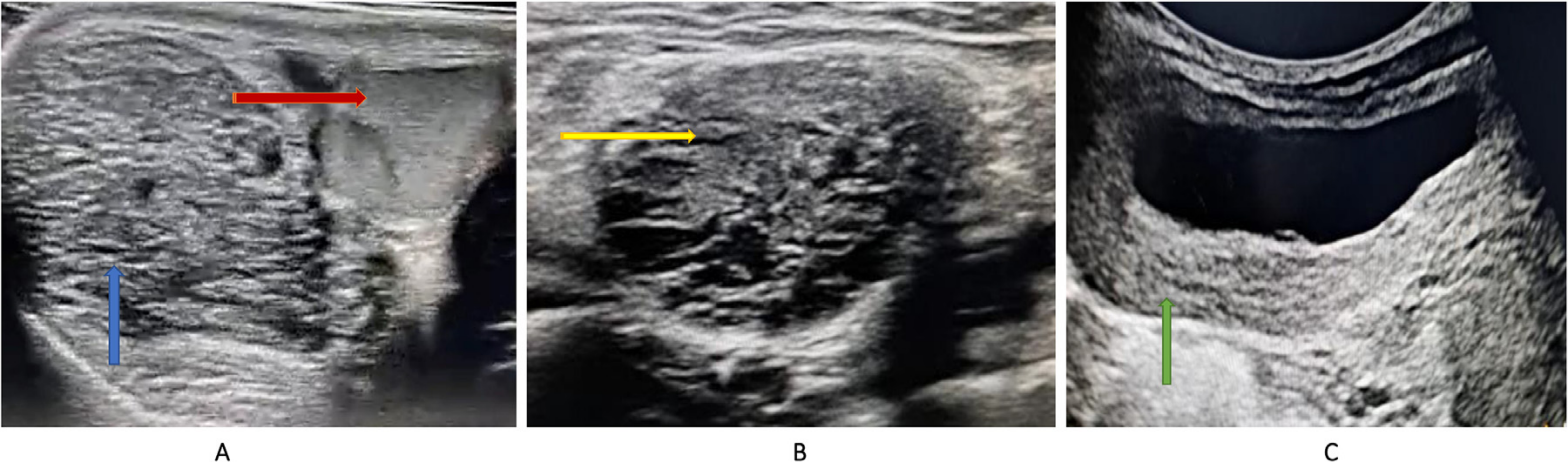
Abdominopelvic ultrasound: • Axial scrotal section (A): Intrascrotal formation (Blue arrow), extratesticular (Red arrow), roughly oval in shape, hypoechoic, heterogeneous, with a microcystic appearance, and traversed by horizontal trabeculae. • Axial pelvic section (B): Hypertrophy of the spermatic cord (Yellow arrow), with a heterogeneous hypoechoic content, containing nonvascularized cystic formations on color Doppler imaging. • Axial pelvic section (C): Regular and diffuse bladder wall thickening, not displaying on color Doppler (Green arrow).

**Fig. 3:**
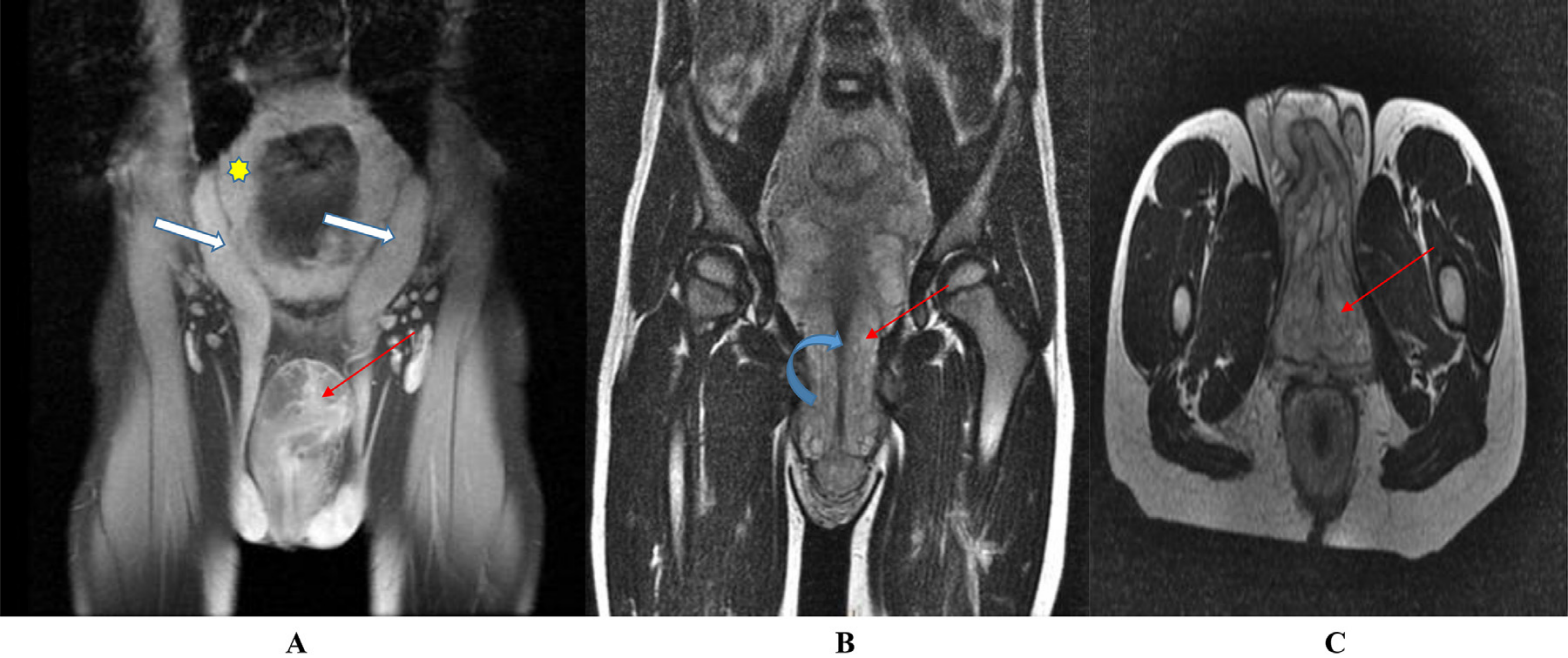
Pelvic MRI in the coronal T2 plane (A) and T1 (B), and in the axial T1 plane (C). **•** The spermatic cords (White arrows) (A) are enlarged, exhibiting diffuse and homogeneous infiltration with high T2 signal intensity. **•** Diffuse bladder wall thickening (Yellow star) (A). • Pelvic infiltration with extension towards the scrotal envelopes, infiltrating the membranous and spongy urethra (Blue arrow) and separating the corpora cavernosa, resulting in the thickened appearance of the penis (Red line) (B) (C).

An ultrasound-guided biopsy of the bladder thickening was performed, and histopathological examination confirmed the presence of bladder neurofibromatosis, also suggesting associated genital involvement. The child is being closely monitored, with no complaints during follow-up visits (Figs. 2 and 3).

## Discussion

We report a case of urogenital involvement in Von Recklinghausen disease in a 10-year-old child, initially detected by scrotal ultrasound and confirmed by pelvic MRI, which allowed the assessment of other pelvic locations of the disease.

Urogenital involvement in NF1 is poorly understood and uncommon [4]. Few cases are described in the literature. The bladder remains the most frequently affected organ. Other organs may also be involved, such as the external genitalia (penis, clitoris, testes), urethra, ureter, and spermatic cord [5].

The first cases of bladder neurofibromas were reported by Smith in 1849 and Gerhardt in 1879, and the first case of bladder neurofibromatosis in a child was reported by Kass in 1932, with a male predominance and a sex ratio of 3 [5,6]. Anatomically, bladder neurofibromas arise from the vesicoprostatic plexus in boys and from the uro-vaginal plexus in girls [5]. They may be isolated or associated with genital involvement. Rink and Mitchell noted that 35% of bladder neurofibromas were associated with genital involvement [7,8], as was the case in our patient.

To our knowledge, only 15 cases of neurofibromas of the male genital tract have been described in the literature. Among these, 9 were neurofibromas of the spermatic cord, 5 of which were intra-scrotal [[9], [10], [11], [12], [13]].

Clinical manifestations are variable, with café-au-lait spots being the pathognomonic sign (>5). However, these spots are only found in 40% of cases after puberty. Bladder involvement may present with symptoms such as dysuria, frequent urination, acute urinary retention, pelvic mass, abdominal pain, and/or recurrent urinary tract infections [14], or it may be asymptomatic, as in our patient's case, who had no urinary symptoms or impact on the upper urinary tract. Intrascrotal involvement, penile, and spermatic cord neurofibromas may present as a palpable mass, dull pain, or loss of sensation. Our patient's presentation included scrotal and penile swelling associated with a nontender palpable mass in the bilateral inguinal region without any inflammatory signs.

Macroscopically, these tumors are sometimes well-defined, but may also present a more diffuse and infiltrative character. The neurofibroma is composed of Schwann cells, perineural-like cells, and fibroblasts. Those developed along a nerve pathway present a fusiform appearance with thickened epineurium on the periphery [15]. The absence of a clear boundary and encapsulation differentiates them from schwannomas. Histologically, the tumor cells are organized into bundles within a myxoid stroma, associated with varying amounts of collagen fibers.

Ultrasound is the modality of choice for any progressive scrotal swelling, and in NF1 patients, it can show multiple hypoechoic masses along the distribution of peripheral nerves [16]. Ultrasound can also exclude tumors or high-flow vascular malformations.

MRI is the method of choice for diagnosing and monitoring neurofibromas [17]. It was also selected for our patient. It helps determine the diagnosis, morphology, type, size of the lesion, extent of the disease, and differentiate it from other very similar lesions such as lymphatic or venous malformations and hemangiomas. MRI is particularly useful in differentiating it from a malignant peripheral nerve sheath tumor [18]. On T1-weighted images, it appears iso-intense compared to muscle tissue. On T2-weighted images, it appears as a homogeneous hyperintense image with a “target sign” due to the central fibrous tissue surrounded by myxoid tissue [17]. They are best seen on T2-weighted images with fat suppression as confluent multinodular masses along the distribution of peripheral nerves [16].

Management of neurofibromatosis varies depending on factors such as size, shape, extent of involvement, clinical symptoms, and impact.

Surgery is indicated if the mass becomes large in adulthood, and reduction surgery may be necessary. The prognosis for neurofibromas is generally good, with rare malignant degeneration [5].

## Conclusion

Urogenital involvement in neurofibromatosis remains infrequent and poorly understood. It affects the pediatric population, with clinical manifestations varying, while imaging aids in diagnosis, and treatment depends on several factors.

## Patient consent

An informed consent was obtained from the kid's parents.

## References

[1] Boyd K.P., Korf B.R. (2009). Theos A : neurofibromatose de type 1. J Am Acad Dermatol.

[2] Poyhonen M., Kytola S., Leisti J. (2000). Épidémiologie de la neurofibromatose de type 1 (NF1) dans le nord de la Finlande. J Med Genet.

[3] Korf B.R. (1999). Neurofibromes plexiformes. Am J Med Genet.

[4] Ameur A., Touiti D., Jira H., Alami M.E., Ouahbi Y., Abbar M. (2003). Aspects urogénitaux et néphrologiques de la maladie de Von Recklinghaussen. A propos de deux observations et d'une revue de la littérature. Annales d'Urologie.

[5] El Ounani F., Dafiri R. (2010). Imagerie de la neurofibromatose vésicale. J de Radiologie.

[6] Ure I., Gurocak S., Gonul I.I., Sozen S., Deniz N. (2013). Neurofibromatosis type I with bladder involvement. Case Rep Urol.

[7] Rink R.C., Mitchell M.E. (1983). Genitourinary neurofibromatosis in childhood. J Urol.

[8] Pascual-Castroviejo I., Lopez-Pereira P., Savasta S., Lopez-Gutierrez J.C., Lago C.M., Cisternino M. (2008). Neurofibromatosis type 1 with extrernal genitalia involvement presentation of 4 patients. J Pediatr Surg.

[9] Levant B., Chetlin M.A. (1948). Neurofibroma of the tunica albuginea testis. J Urol.

[10] Livolsi V.A., Schiff M. (1948). Myxoid neurofibroma of the testis. J Urol.

[11] Yamamoto M., Miyake K., Mitsuya H. (1982). Intrascrotal extratesticular neurofibroma. Urology.

[12] Yoshimura K., Maeda O., Saiki S., Kuroda M., Miki T., Usami M. (1990). Solitary neurofibroma of the scrotum. J Urol.

[13] Gupta S., Gupta R., Singh S., Pant L. (2011). Solitary intrascrotal neurofibroma: a case diagnosed on aspiration cytology. Diagn Cytopathol.

[14] Gao B., De Cotiis K., Bobrowski A., Koyle M., O'Kelly F. (2020). The association of neurofibromatosis type I and lower urinary tract dysfunction in the paediatric population – a critical review of literature. J Pediatr Urol.juin.

[15] Bouvier C., Maues de Paula A., Roche P.-H., chagnaud C., Figarella-Branger D. (2013). Tumeurs du système nerveux périphérique. EMC-Neurologie Janv.

[16] Sereni C.P., Rodgers S.K. (2015). Cas SRU du jour : neurofibrome plexiforme. Ultrasound Q.

[17] Souza F.H., Dabdoub C.B., Bernardino S.N. (2013). Neurofibromes plexiformes bilatéraux des plexus brachial et lombo-sacré. Arq Neuropsiquiatr.

[18] Lim C., Kwon K., Lee K. (2014). Neurofibrome plexiforme traité par pharmacopuncture. J Pharmacopuncture.

